# *Annona muricata* Polyphenols Induce Cytoskeletal Alterations and Predicted Aldose Reductase Binding in *Giardia lamblia*

**DOI:** 10.1155/adpp/4133207

**Published:** 2026-08-25

**Authors:** Diana Valeria Govea-Flores, Mariana Garza-Ontiveros, Filiberto Gutiérrez-Gutiérrez, Ana Gabriela Gómez-Ibarra, Sendar Daniel Nery-Flores, Juan Alberto Ascacio-Valdés, Lizeth Guadalupe Campos-Múzquiz, Raúl Rodríguez-Herrera, Lissethe Palomo-Ligas

**Affiliations:** ^1^ Faculty of Chemical Sciences, Autonomous University of Coahuila, Saltillo Campus, Saltillo Coahuila, 25280, Mexico, uadec.mx; ^2^ Department of Pharmacobiology, University Center for Exact Sciences and Engineering, University of Guadalajara, Guadalajara Jalisco, 44430, Mexico, udg.mx

**Keywords:** aldose reductase, phytochemicals, protozoa, soursop, tubulin

## Abstract

Giardiasis has been classified as a neglected disease by the World Health Organization since 2004. Despite ongoing efforts, challenges such as drug resistance and treatment failures continue to hinder effective management. In recent years, the exploration of plant‐derived compounds as antiparasitic agents has emerged as a promising strategy. This is supported by the discovery of plant‐derived compounds such as quinine and artemisinin in antiparasitic pharmacology. The identification of specific molecular targets in *Giardia lamblia*, a protozoan with unique cytoskeletal and metabolic features, has further advanced drug discovery efforts.

In this study, we evaluated the in vitro and in silico effects of a polyphenol‐enriched extract from *Annona muricata* (soursop) leaves against *G. lamblia* trophozoites. The extract had a noticeable impact on parasite growth, cell adhesion, and morphology. Ultrastructural analyses revealed elongation, flagellar abnormalities, and damage to the ventral disk. Western blot and immunofluorescence assays demonstrated reduced *α*‐tubulin expression and altered localization, indicating cytoskeletal alterations in treated trophozoites. Molecular docking analyses predicted favorable interactions between soursop polyphenols and *Giardia* aldose reductase, particularly involving the catalytic residues Y40 and K71. Collectively, these findings suggest that *A. muricata* polyphenols exert antigiardial effects through multiple cellular alterations and support the hypothesis that metabolic and cytoskeletal processes may both contribute to the observed biological effects. Further studies are required to experimentally validate the role of aldose reductase and its relationship with cytoskeletal disruption.

## 1. Introduction

Giardiasis is an intestinal infection caused by the protozoan *Giardia lamblia*. Transmission occurs primarily through cyst ingestion via contaminated water, food, or hand‐to‐mouth contact [1]. After ingestion, exposure to the acidic environment of the stomach triggers excystation in the duodenum, releasing trophozoites, the active and replicative form of the parasite. These trophozoites colonize the small intestine by binary fission and may lead to clinical manifestations such as diarrhea. In response to environmental changes (e.g., pH and bile concentration), trophozoites encyst and are excreted in feces. The cyst form is environmentally resistant, allowing continued transmission to new hosts [2].

Giardiasis is a globally distributed disease, with a high prevalence in developing countries. Its incidence is closely linked to poor hygiene, inadequate water sanitation, and overcrowded living conditions [3]. Children under 5 years old are particularly vulnerable and often experience stunting, wasting, and underweight due to chronic infections [4].

Current treatment relies primarily on 5‐nitroimidazole compounds; however, increasing resistance to these drugs has been reported [5]. Multiple factors contribute to treatment failure, including parasite‐related issues (multidrug‐resistant strains and reinfection), drug‐related factors (treatment adherence and pharmacokinetic variability), and host‐related variables such as microbiota composition and immune status [6].

To support existing therapies, the search for new antigiardial agents from natural sources remains a promising alternative [7–9]. In this context, plants of the *Annona* genus have attracted increasing attention due to their traditional medicinal use and documented antiparasitic activity [10, 11]. *Annona muricata* L., commonly known as soursop, is a tropical and subtropical plant traditionally used to treat gastrointestinal disorders, including dysentery and parasitic infections [12]. Its antiparasitic potential has been reported against *Toxoplasma gondii*, *Plasmodium falciparum* [13, 14], and *Leishmania* species [15]. Similarly, *Annona cherimola* has demonstrated in vitro antiprotozoal activity against *Entamoeba histolytica* and *G. lamblia* [16]. More recently, peptides extracted from *Annona diversifolia* Saff. were shown to exhibit giardicidal activity, with molecular docking analyses indicating strong interactions with the parasite’s aldose reductase and PFOR enzymes [17].

From a phytochemical perspective, flavonoids are among the most abundant classes of secondary metabolites in the *Annona* genus, with quercetin, luteolin, kaempferol, catechin, and rutin being some of the predominant compounds [18]. These compounds are widely recognized for their biological activities and their ability to interact with key cellular targets in parasites [19–22].

While previous studies on antigiardial compounds have primarily focused on cytoskeletal proteins such as *α*‐tubulin as pharmacological targets [23, 24], increasing evidence suggests that metabolic enzymes may also contribute to parasite susceptibility [25–28]. Among these enzymes, aldose reductase has attracted attention because of its involvement in carbohydrate metabolism and redox homeostasis. In *Giardia*, aldose reductase participates in acetaldehyde metabolism and ethanol production through an NADPH‐dependent pathway, contributing to intracellular redox balance [29].

Flavonoids such as kaempferol and its derivatives, commonly identified in *Annona* species, have been reported as aldose reductase inhibitors in other biological systems [30, 31]. Moreover, previous docking studies suggested that kaempferol may interact with the *Giardia* homolog of this enzyme [32]. Since aldose reductase inhibition has also been associated with alterations in tubulin polymerization and cytoskeletal organization [33, 34], it is possible that metabolic and structural pathways may be functionally connected in *Giardia* trophozoites.

Therefore, the aim of this study was to evaluate the antigiardial activity of a polyphenol‐enriched fraction obtained from *A. muricata* leaves and to investigate potential mechanisms associated with its activity. Aldose reductase was selected as a putative molecular target based on its role in *G. lamblia* carbohydrate metabolism and redox homeostasis, as well as previous evidence supporting its susceptibility to flavonoid‐type inhibitors. To address this objective, trophozoite growth, adherence, apoptosis‐like cell death, and ultrastructural alterations were evaluated in vitro. In addition, changes in *α*‐tubulin expression and distribution were analyzed to assess cytoskeletal alterations. Finally, molecular docking analyses were performed to predict the interaction between the identified polyphenols and *Giardia* aldose reductase as a potential molecular target. These computational analyses were intended to provide mechanistic hypotheses that may contribute to explaining the observed biological effects.

## 2. Material and Methods

### 2.1. Preparation of Ethanol Extract and Polyphenol‐Enriched Fraction of Soursop Leaf

Soursop leaves were acquired commercially from a specialized company in Las Varas. Nayarit, Mexico [35]. The vegetal sample was cleaned, dehydrated, and reduced to a particle size of 0.6 mm. The preparation of the ethanol extract was performed by a microwave/ultrasound hybrid methodology in an Ultrasonic Microwave Reaction System (Nanjing ATPIO Instruments Manufacture Co. Ltd., Nanjing, China) using a relation of 1:12 (mass/volume ratio). For the ultrasound‐assisted extraction, the following parameters were used: power ratio 20, ultrasonic on relay 10, ultrasonic off relay 3, amplitude transformer 25, and set 20. Microwave‐assisted extraction was performed using a power ratio of 800, display power of 0, set temperature of 70°C, and a holding time of 5 min. After treatment, the ethanol extract was filtered using Whatman No. 41 filter paper (Cytiva, Uppsala, Sweden). The polyphenolic‐rich fraction was obtained by column chromatography using Amberlite XAD‐16 resin (MilliporeSigma, Darmstadt, Germany) as the stationary phase and ethanol as eluent. These procedures were performed according to the specifications of a previous report [36, 37].

### 2.2. Phytochemical Profiling by Folin–Ciocalteu, Flavonoid Quantification, and HPLC/MS

Phytochemical profiling of the polyphenol‐enriched fraction obtained from *A. muricata* (soursop) leaves was performed using colorimetric assays and chromatographic analysis. Total hydrolyzable polyphenols were determined using the Folin–Ciocalteu method, as previously described [38]. Briefly, 15 μL of the sample (1 mg/mL) was dispensed in triplicate into a 96‐well microplate, followed by the addition of 170 μL of Milli‐Q water and 12 μL of Folin–Ciocalteu reagent. After incubation for 5 min at room temperature, 30 μL of Na_2_CO_3_ solution (200 g/L) was added, and the plate was incubated in the dark for 1 h at room temperature. Subsequently, 73 μL of Milli‐Q water was added, and absorbance was measured at 765 nm using a spectrophotometer (Epoch BioTek ELISA plate reader). Gallic acid (Sigma‐Aldrich; G7384) was used to generate the calibration curve, and results were expressed as milligrams of gallic acid equivalents per gram of dry extract (mg GAE/g).

Total flavonoid content was quantified by the aluminum chloride complexation assay under alkaline conditions, following the protocol described by Aguirre‐García et al. [38]. Briefly, 200 μL of the sample (1 mg/mL) was mixed with 280 μL of Milli‐Q water and 60 μL of 5% NaNO_2_ and incubated for 5 min at room temperature. Then, 60 μL of 10% AlCl_3_ was added, and the reaction was incubated for 10 min in the dark. Subsequently, 400 μL of 1 M NaOH was added, and 200 μL of the reaction mixture was transferred in triplicate to a 96‐well microplate. Absorbance was measured at 510 nm. Catechin (Sigma‐Aldrich; C1251) was used as the reference standard, and results were expressed as milligrams of catechin equivalents per gram of dry extract (mg CE/g).

The polyphenol‐enriched fraction was further analyzed by reverse‐phase high‐performance liquid chromatography coupled with mass spectrometry (HPLC/MS). The sample was dissolved in ethanol at a concentration of 2 mg/mL and filtered through 0.45 μm nylon membranes before analysis. Chromatographic separation was performed on a C18 column using a Varian ProStar system (Agilent Technologies, Santa Clara, CA, USA) equipped with a photodiode array detector set at 280 nm. The mobile phases consisted of Solvent A (0.2% formic acid in water) and Solvent B (acetonitrile). A gradient elution was applied as follows: 0–5 min, 3%–9% B; 5–15 min, 9%–16% B; 15–45 min, 16%–50% B. The flow rate was maintained at 0.2 mL/min, and the column temperature was set at 30°C. The whole effluent was directly introduced into the mass spectrometer without splitting. Mass spectrometric detection was carried out on a Varian 500/MS ion trap mass spectrometer (Agilent Technologies) using electrospray ionization in negative mode ([M–H]^−^). The ion source parameters included a capillary voltage of 90 V, a spray voltage of 5.0 kV, and a temperature of 350°C. Data were acquired in full scan mode over an *m*/*z* range of 50–2000. Compound identification was achieved by comparison with a database of bioactive compounds (WorkStation Version 2.0, Varian) and previously reported data [39]. To estimate the relative content of the identified polyphenols, chromatographic peak areas were calculated by measuring the area under the curve (AUC) using GraphPad Prism 6. The abundance of each analyte was expressed as relative peak area (%) [37].

### 2.3. Culture and Growth Inhibition Assay of *G. lamblia* Trophozoites

*G. lamblia* trophozoites (ATCC 50803) were cultured in TYI‐S‐33 medium (pH 7.0), supplemented with 10% bovine serum and 0.5 mg/mL bovine bile, as previously described [40]. Cultures were maintained by subculturing twice weekly. For all experiments, trophozoites in the logarithmic phase were detached by incubation in an ice‐water bath for 30 min and counted using a hemocytometer (Propper Manufacturing Co., USA). To evaluate the effect of soursop polyphenols (SPP) on trophozoite growth, 3.5 × 10^5^ cells were cultivated per tube and treated with SPP at concentrations of 150, 175, or 200 μg/mL. The solvent control consisted of 0.1% dimethyl sulfoxide (DMSO; MilliporeSigma, Darmstadt, Germany), while metronidazole (MTZ, 1.5 μg/mL) was included as a reference drug [37]. All cultures were incubated at 37°C for 24, 48, or 72 h. After each time point, cells were collected by chilling in an ice‐water bath and counted with a hemocytometer (Propper Manufacturing Co., USA). Trophozoite growth was expressed as a percentage relative to the DMSO‐treated control.

### 2.4. Effect on Adhesion Capacity and Apoptosis or Necrosis‐Like Cell Death in *Giardia*

To evaluate the effect on cell adhesion, *G. lamblia* trophozoites were exposed to SPP at concentrations of 150, 175, and 200 μg/mL, as well as to a positive control using albendazole (ABZ; 0.133 μg/mL; MilliporeSigma, Darmstadt, Germany). At the end of each incubation period (24, 48, and 72 h), adhered and non‐adhered cells were quantified. Free (non‐adhered) trophozoites were first collected in microtubes, and the adhered population was recovered by rinsing the culture tubes with phosphate‐buffered saline (PBS). Both cell fractions were cooled in an ice‐water bath for 30 min and counted using a hemocytometer (Propper Manufacturing Co., USA). Results were expressed as the percentage of cell adhesion inhibition relative to DMSO‐treated control.

For the immunofluorescent detection of cell death processes in *Giardia* trophozoites, Annexin V‐FITC and propidium iodide (PI) staining were used to identify apoptotic and necrotic‐like cells, respectively. The Annexin V‐FITC Apoptosis Detection Kit (Abcam, ab14085) was used according to the manufacturer’s instructions with minor modifications. Trophozoites treated for 48 h with 0.1% DMSO (vehicle), SPP (150–200 μg/mL), or ABZ (0.133 μg/mL) were detached by incubation in an ice‐water bath for 45 min. A total of 1 × 10^6^ cells were collected by centrifugation and washed twice with PBS. The cell pellet was resuspended in 250 μL of binding buffer and stained with 5 μL of each fluorescent dye for 5 min in the dark. Samples were visualized using an Axiolab A1 35 LED fluorescence microscope (Carl Zeiss™), and the number of Annexin V‐ or PI‐positive cells was counted using a hemocytometer (Propper Manufacturing Co., USA) [41]. Results were expressed as the percentage of stained cells in each marker.

### 2.5. Scanning Electron Microscopy (SEM) Analysis in *Giardia* Trophozoites

Trophozoites were cultured for 48 h in the presence of soursop polyphenolic extract or 0.1% DMSO as a vehicle control. After incubation, cells were detached using an ice‐water bath and collected by centrifugation. Parasites were washed with PBS and allowed to adhere to glass coverslips pretreated with 0.2% polyethylenimine (MilliporeSigma, Darmstadt, Germany). For fixation, samples were incubated with 2.5% glutaraldehyde (MilliporeSigma, Darmstadt, Germany) for 1 h at room temperature. Dehydration was carried out through a graded ethanol series (50%–100% v/v), followed by critical point drying with CO_2_. Finally, the specimens were sputter‐coated with a thin layer of gold and examined using a field emission scanning electron microscope (FE‐SEM; Hitachi High‐Technologies Corporation, Tokyo, Japan) [36].

### 2.6. In Silico Analysis of Polyphenols Binding to *G. lamblia* Aldose Reductase by Molecular Docking

To predict potential molecular interactions between polyphenolic compounds from *A. muricata* extract and *G. lamblia* aldose reductase enzyme, molecular docking simulations were performed. The chemical structures of each polyphenol were optimized using the MMFF94 force field in Avogadro software [42]. Atomic partial charges were assigned using the Gasteiger–Marsili method [43], and both ligand and receptor preparation—including addition of polar hydrogens and assignment of atom types—were performed using MGLTools v1.5.6 [44].

Docking simulations were conducted using AutoDock software [44]. The three‐dimensional structure of *G. lamblia* aldose reductase (PDB: 3KRB) was used as the receptor. A grid box of 15 Å^3^ was centered on the reported inhibitor binding site at coordinates (25.762, 39.00, and 16.49). For each ligand, 20 binding modes were generated with a maximum of 25,000,000 energy evaluations to achieve higher conformational resolution at the binding site. The resulting binding conformations were clustered based on root‐mean‐square deviation (RMSD) with a threshold of < 2 Å [45]. The most favorable ligand–enzyme complexes were selected based on the lowest binding energy scores (kcal/mol). Visualization and structural analysis were performed using Maestro (Schrödinger Release 2017‐1, Schrödinger, LLC, New York, NY, USA).

### 2.7. Docking Protocol Validation

To validate the docking parameters, we used the crystal structure of aldose reductase co‐crystallized with the known inhibitor IDD‐594 (PDB ID: 2R24) from *Rattus norvegicus*. Although this structure belongs to a different species, a structural alignment of the monomeric forms (excluding water molecules) between 2R24 and the *G. lamblia* aldose reductase (PDB ID: 3KRB) showed high conservation of the core fold, with an RMSD of 0.605 Å. Therefore, validation on this homologous enzyme is justified. The known inhibitor IDD‐594 was re‐docked into its own binding site using AutoDock 4.2.6 with the same parameters described above. The resulting pose was compared to the co‐crystallized conformation; an RMSD < 2 Å was obtained, confirming that the docking protocol reliably reproduces the experimental binding mode. These validated parameters were then applied to dock the polyphenolic compounds into the *G. lamblia* aldose reductase structure (PDB ID: 3KRB), for which no inhibitor‐bound structure is available.

### 2.8. Determination of *α*‐Tubulin Expression in *G. lamblia* Trophozoites

Trophozoites exposed to SPP for 48 h were detached and collected by centrifugation. Cell lysates were prepared using NP‐40 lysis buffer supplemented with a protease inhibitor cocktail (Roche, Basel, Switzerland), and protein concentrations were determined using the BCA Protein Assay Kit (Thermo Fisher Scientific, Waltham, MA, USA). For SDS–PAGE, 10 μg of total protein per sample was separated on a 10% (v/v) polyacrylamide gel using Tris–glycine running buffer. Proteins were transferred to PVDF membranes (Millipore, Burlington, VT, USA) using a Trans‐Blot® SD Semi‐Dry Transfer Cell (Bio‐Rad Laboratories, Hercules, CA, USA). Membranes were blocked with nonfat dry milk (Santa Cruz Biotechnology, Dallas, TX, USA) for 90 min at room temperature. The *α*‐tubulin detection was performed using a mouse monoclonal primary antibody (sc‐23948, Santa Cruz Biotechnology) at a 1:500 dilution, followed by incubation with an HRP‐conjugated secondary antibody (sc‐516102, Santa Cruz Biotechnology) at a 1:5000 dilution. Signal development was carried out using the Immobilon ECL Ultra Western HRP Substrate Kit (Millipore).

Bands were visualized and captured using VisionWorks software (v8.17.16133.9147, Analytik Jena). Relative *α*‐tubulin expression was quantified by densitometric analysis using GelQuant.NET software (biochemlabsolutions.com). *α*‐Tubulin levels were determined by integrated optical density (IOD) and normalized to the total protein content per lane, assessed by Coomassie Blue staining of the membrane, as recommended for Western blot normalization to avoid housekeeping protein bias [46]. Experiments were performed in three independent biological replicates, and each band was quantified in quintuplicate [41].

### 2.9. Immunofluorescence Analysis of *α*‐Tubulin Localization in *Giardia* Trophozoites

Trophozoites exposed for 48 h to either 0.1% DMSO or SPP were harvested and washed with PBS. Cells were adhered for 30 min at 37°C onto coverslips pretreated with 0.2% polyethylenimine (MilliporeSigma, Darmstadt, Germany) and fixed with absolute methanol at −20°C for 10 min. Permeabilization was carried out using 0.5% Triton X‐100 for 15 min (MilliporeSigma, Darmstadt, Germany), followed by blocking with 1% bovine serum albumin (BSA) for 1 h at room temperature.

Detection of *α*‐tubulin was performed using a mouse monoclonal IgG1 *κ* anti‐*α*‐tubulin primary antibody (sc‐23948, Santa Cruz Biotechnology, Dallas, TX, USA) diluted 1:200, and a fluorescein isothiocyanate (FITC)‐conjugated secondary antibody (sc‐516140, Santa Cruz Biotechnology) also at 1:200 dilution. Nuclei were counterstained with ProLong™ Gold Antifade Mountant with DAPI (Thermo Fisher Scientific, Waltham, MA, USA). Samples were visualized using an Axiolab A1 35 LED Binocular Fluorescence Microscope (Carl Zeiss™, Oberkochen, Germany), and images were captured with ZEN 2.3 software [36].

### 2.10. Statistical Analysis

All statistical analyses were performed using GraphPad Prism Version 6 (GraphPad Software, San Diego, CA, USA). Statistical significance was established at *p* < 0.0 5. Data were expressed as mean ± standard deviation (SD), and significant differences were indicated relative to the corresponding control group in each figure. IC_50_ values were determined by nonlinear regression analysis of dose–response curves using GraphPad Prism.

Effects on growth, cell adherence, and apoptosis‐like and necrotic cell death data were analyzed using two‐way analysis of variance (ANOVA) followed by Tukey’s post hoc test. For Western blot analysis, normalized protein expression values and IOD measurements were analyzed using one‐way ANOVA followed by Tukey’s post hoc test.

## 3. Results

### 3.1. Phytochemical Profiling: Total Phenolics, Flavonoids, and HPLC/MS Analysis

To characterize the chemical composition of the *A. muricata* leaf polyphenol‐enriched fraction and establish its phytochemical composition, we first quantified total phenolic and flavonoid contents and subsequently performed HPLC/MS‐based compound identification.

The phytochemical characterization of the polyphenol‐enriched fraction obtained from *A. muricata* leaves revealed a high content of bioactive compounds. After purification using Amberlite XAD‐16 resin, the extraction yield of the polyphenol‐enriched fraction was 2.74%. Quantitative colorimetric analysis showed that the extract contained 242.4 ± 5.7 mg gallic acid equivalents per gram of dry extract (mg GAE/g) of hydrolyzable polyphenols, along with 101.9 ± 5.5 mg catechin equivalents per gram of dry extract (mg CE/g) of total flavonoids.

Further characterization by HPLC/MS analysis confirmed the presence of flavonoids in the polyphenol‐enriched fraction based on the identified compounds (Table 1, Figure S1). The analysis revealed the presence of five polyphenolic compounds, and relative quantification based on chromatographic peak areas enabled estimation of their abundance. Apigenin 6,8‐di‐C‐glucoside was identified as the predominant compound, representing 55.80% of the total relative peak area. This was followed by two glycosylated kaempferol derivatives with distinct sugar substitutions: kaempferol 3‐O‐galactoside 7‐O‐rhamnoside (17.30%) and kaempferol 3,7‐O‐diglucoside (11.85%). Lower relative abundances were observed for (+)‐catechin (10.14%) and cyanidin 3,5‐O‐diglucoside (4.83%).

**TABLE 1:** Compounds identified by HPLC/MS in a polyphenol‐enriched fraction of *Annona muricata* (soursop) leaf ethanol extract.

Identity	Family	Retention time (min)	m/*z* ([M‐H]^−^)	Relative peak area (%)
Cyanidin 3,5‐O‐diglucoside	Anthocyanins	19.046	612	4.83
(+)‐Catechin	Catechins (flavan‐3‐ols)	21.367	289	10.14
Kaempferol 3,7‐O‐diglucoside	Flavonols	28.508	609.2	11.85
Apigenin 6,8‐di‐C‐glucoside	Flavones	30.174	593.2	55.80
Kaempferol 3‐O‐galactoside 7‐O‐rhamnoside	Flavonols	31.362	593.2	17.30

*Note:* Data were analyzed with WorkStation software v2.0 (VARIAN) and GraphPad Prism 6.

### 3.2. The Growth of *Giardia* Trophozoites Was Diminished by SPP

**FIGURE 1:**
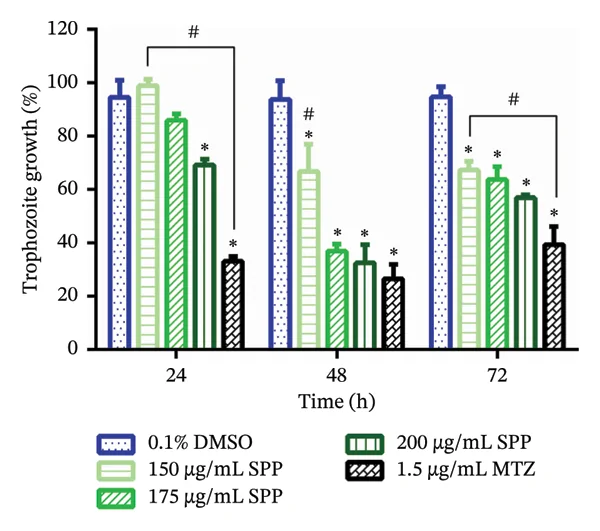
Soursop polyphenols (SPP) inhibit *Giardia lamblia* trophozoite growth in vitro. Parasite growth is expressed as a percentage relative to the DMSO control. Data are presented as mean ± SD from four independent biological experiments (*n* = 4). Treatment with SPP reduced parasite growth at the evaluated time points, with the greatest effect observed at 48 h. Statistical analysis was performed using two‐way ANOVA followed by Tukey’s post hoc test. ^∗^ *p* < 0.05 versus DMSO; ^#^ *p* < 0.05 versus MTZ.

To evaluate the effect of the *A. muricata* polyphenol‐enriched fraction on *G. lamblia* proliferation, growth kinetics assays were performed in trophozoites. SPP reduced the growth of *G. lamblia* trophozoites in a concentration‐dependent manner (Figure 1). After 24 h of exposure, a significant effect was observed only at 200 μg/mL, where parasite growth decreased to 69.25% of the control, corresponding to a growth inhibition of 30.75% and an estimated IC_50_ of 180.6 μg/mL (log_10_ IC_50_ = 2.257). The strongest effect was detected at 48 h, when all tested concentrations significantly reduced parasite growth, yielding an IC_50_ of 159.0 μg/mL (log_10_ IC_50_ = 2.202). At this time point, the lowest growth value was observed at 200 μg/mL (32.56% of the control), corresponding to a maximum inhibition of 67.44%. After 72 h, parasite growth was 67.36%, 63.71%, and 56.92% for 150, 175, and 200 μg/mL, respectively, resulting in an estimated IC_50_ of 186.8 μg/mL (log_10_ IC_50_ = 2.271). Although growth remained significantly lower than in the control group, a slight increase in parasite numbers was observed at 175 and 200 μg/mL compared with the 48 h time point. MTZ showed the greatest inhibitory effect throughout the experiment and was significantly more active than SPP, except at 48 h, when its effect was comparable to those observed with 175 and 200 μg/mL.

### 3.3. SPP Impair Cell Adhesion and Induce Apoptosis‐Like Death in *G.lamblia*

**FIGURE 2:**
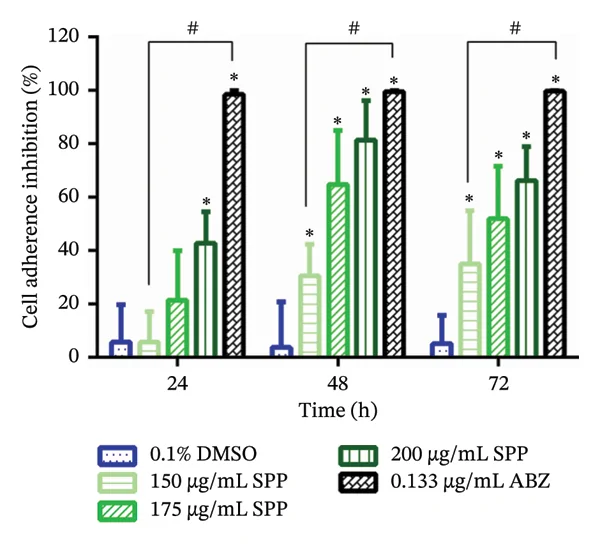
Soursop polyphenols (SPP) impair the adhesion capacity of *Giardia lamblia* trophozoites. Cell adhesion inhibition is expressed as a percentage, and values represent the mean ± SD of four independent biological experiments (*n* = 4). Treatment with SPP reduced trophozoite adhesion at the evaluated time points, with the greatest effect observed at 48 h. Statistical analysis was performed using two‐way ANOVA followed by Tukey’s post hoc test. ^∗^ *p* < 0.05 versus DMSO; ^#^ *p* < 0.05 versus MTZ.

To determine whether treatment affected trophozoite attachment, adhesion assays were performed to quantify the percentage of adhesion inhibition. SPP treatment also reduced the adhesion capacity of *Giardia* trophozoites, showing a pattern like that observed in the growth kinetics. At 24 h, the highest concentration (200 μg/mL) produced a cell adhesion inhibition of 42.86%. The maximal inhibitory effect occurred at 48 h across all tested concentrations: 30.56% (150 μg/mL), 64.81% (175 μg/mL), and 81.48% (200 μg/mL). By 72 h, the inhibition remained concentration‐dependent but was less pronounced, with significant (*p* < 0.05) reductions of 35.06%, 51.95%, and 66.23%, indicating a slight attenuation of the effect over time. The positive control, ABZ, resulted in greater than 98% inhibition of parasite adhesion at all points tested (Figure 2).

**FIGURE 3:**
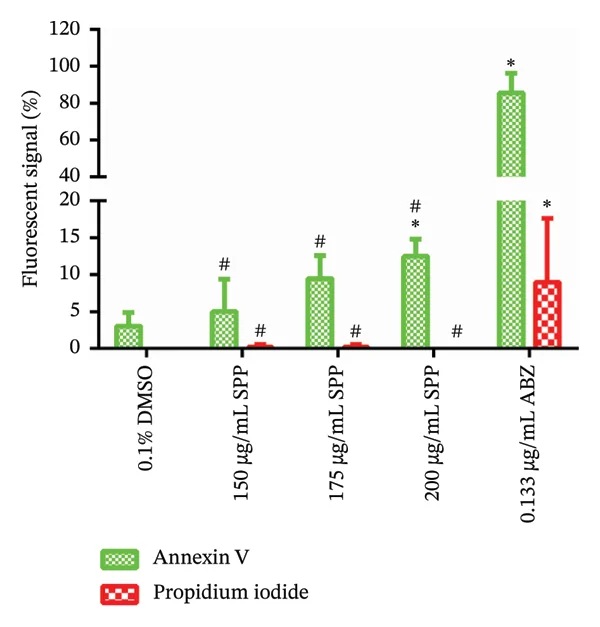
Soursop polyphenols induce apoptosis‐like cell death in *Giardia lamblia* trophozoites. Data are presented as mean ± SD from three independent experiments (*n* = 3). Fluorescence signal intensity for Annexin V and propidium iodide was quantified from at least 100 trophozoites per experiment. Statistical analysis was performed using two‐way ANOVA followed by Tukey’s post hoc test. ^∗^ *p* < 0.05 versus DMSO; ^#^ *p* < 0.05 versus ABZ.

To assess apoptosis‐like events induced by treatment, trophozoites were stained with Annexin V and PI to evaluate phosphatidylserine externalization and membrane integrity. Figure 3 shows that SPP induces apoptosis‐like features in *G. lamblia* trophozoites after 48 h of exposure. Annexin V staining revealed a concentration‐dependent effect, with a significant (*p* < 0.05) increase observed only at 200 μg/mL of SPP. In comparison, ABZ treatment resulted in the highest percentage of Annexin V‐positive cells (85.50%). In contrast, necrosis‐like cell death, as indicated by PI staining, was significantly increased (*p* < 0.05) only in the ABZ‐treated group, with 9.00% PI‐positive cells.

### 3.4. Morphological Alterations in *G. lamblia* Trophozoites Induced by SPP

**FIGURE 4:**
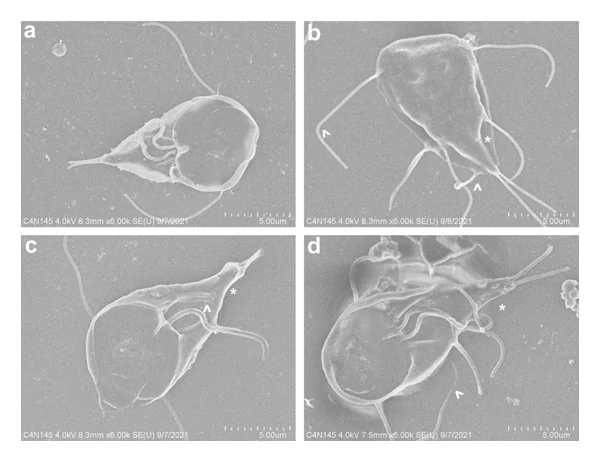
Morphological alterations in *Giardia lamblia* trophozoites induced by soursop polyphenols (SPP). Representative scanning electron micrographs of trophozoites exposed for 48 h to (a) 0.1% DMSO or increasing concentrations of SPP: (b) 150 μg/mL, (c) 175 μg/mL, and (d) 200 μg/mL. Treated cells exhibit concentration‐dependent morphological alterations, including elongation of the caudal region (∗), flagellar disorganization or asymmetry (˄), ventral disk distortion, and membrane blebbing, suggesting cytoskeletal disruption. Scale bar = 5 μm.

To examine treatment‐induced morphological alterations, SEM was used to evaluate the surface and ultrastructural changes in *G. lamblia* trophozoites after 48 h of exposure to SPP. Untreated control cells (0.1% DMSO) maintained the characteristic pyriform shape with organized flagella and an intact ventral disk (Figure 4a). In contrast, trophozoites treated with 150 μg/mL of SPP showed notable elongation of the caudal region (∗), early signs of surface irregularities, and mild flagellar asymmetry (˄) (Figure 4b).

At 175 μg/mL (Figure 4c), these effects became more pronounced, including a marked loss of ventral flagella (˄), distortion of the marginal groove, and enhanced caudal elongation (∗). At the highest tested concentration (200 μg/mL), cells exhibited profound morphological disruption (Figure 4d), with irregular and disorganized flagella (˄), increased surface roughness and membrane blebbing, and evidence of ventral disk collapse. Additionally, some trophozoites exhibited a swollen or rounded appearance. Ultrastructural alterations were more evident at higher SPP concentrations.

### 3.5. Identification of *Giardia* Aldose Reductase as a Potential Molecular Target of SPP

To assess the potential binding interactions underlying the antigiardial activity of SPP, molecular docking studies were conducted using *G. lamblia* aldose reductase as the target enzyme. The results indicate that the AutoDock software is capable of identifying the binding site of *Giardia* aldose reductase inhibitors with good reliability, as demonstrated by the re‐docking validation of the inhibitor IDD‐594, which yielded an RMSD of 1.502 (Figure S2). In addition, the results show that the SPP compounds can bind near the active site of the enzyme, overlapping with the binding site of known inhibitors. Docking scores ranged from −9.9 to −8.2 kcal/mol (Table 2), with kaempferol derivatives, particularly kaempferol 3,7‐O‐diglucoside and kaempferol 3‐O‐galactoside 7‐O‐rhamnoside, exhibiting the highest affinities. These values are slightly lower than the affinity predicted for the reference inhibitor IDD‐594.

**TABLE 2:** Molecular docking scores and protein–ligand interacting residues of soursop polyphenols against *Giardia lamblia* aldose reductase.

Compounds	Docking score (kcal/mol)	Protein–ligand interacting residues
Apigenin 6,8‐di‐C‐glucoside	−8.6	W12, Q13, D35, C36, A37, V39, Y40, K71, W73, H104, F118, Y203, S204, S209, Y210, Q220, C301, F306
(+)‐Catechin	−8.2	W12, Q13, C36, A37, V39, Y40, K71, W73, H104, S155, Q177, Y210
Cyanidin 3,5‐O‐diglucoside	−8.5	W12, Q13, A37, V39, Y40, K71, W73, H104, W105, L117, F118, K120, S209, Y210, Q220, C301, F306, W307
Kaempferol 3,7‐O‐diglucoside	−9.9	W12, Q13, A37, Y38, V39, Y40, Q41, R48, K71, W73, H104, W105, G115, D116, L117, F118, Q177, S204, S209, Y210, C301
Kaempferol 3‐O‐galactoside 7‐O‐rhamnoside	−9.1	W12, Q13, P16, E17, Q20, D35, A37, V39, Y40, Q41, R48, K71, H104, W105, G115, D116, L117, F118, Q177, S204, S209, Y210, C301
IDD 594	−10.8	W12, E17, V39, Y40, Q41, K71, W73, N74, H104, W105, L107, F118, F306, W307

*Note:* Docking score values (kcal/mol) of polyphenols isolated from *Annona muricata* in interaction with the *G. lamblia* aldose reductase enzyme. Lower scores indicate higher binding affinity.

**FIGURE 5:**
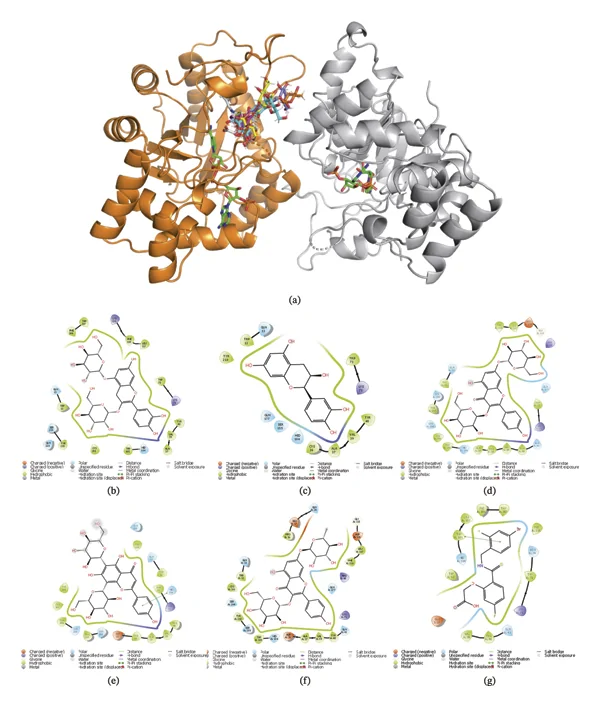
Molecular docking of *Annona muricata* polyphenols with *Giardia lamblia* aldose reductase. (a) Overall binding site within the aldose reductase homodimer. Panels (b–g) depict the predicted binding modes of individual compounds: (b) apigenin 6,8‐di‐C‐glucoside, (c) (+)‐catechin, (d) cyanidin 3,5‐O‐diglucoside, (e) kaempferol 3,7‐O‐diglucoside, (f) kaempferol 3‐O‐galactoside 7‐O‐rhamnoside, and (g) reference inhibitor IDD‐594. All ligands were predicted to bind within the conserved catalytic pocket, overlapping the inhibitor‐binding site.

Interestingly, all tested polyphenols docked at a conserved site on the enzyme (Figure 5a), coinciding with the inhibitor binding region and showing interactions primarily with amino acid residues Y40 and K71 (Figure 5b–g, Table 2). These residues are key components of the catalytic pocket, involved in proton transfer and charge stabilization with nicotinamide and the substrate.

### 3.6. Alteration in *Giardia α*‐Tubulin Expression Level and Immunolocalization by SPP

**FIGURE 6:**
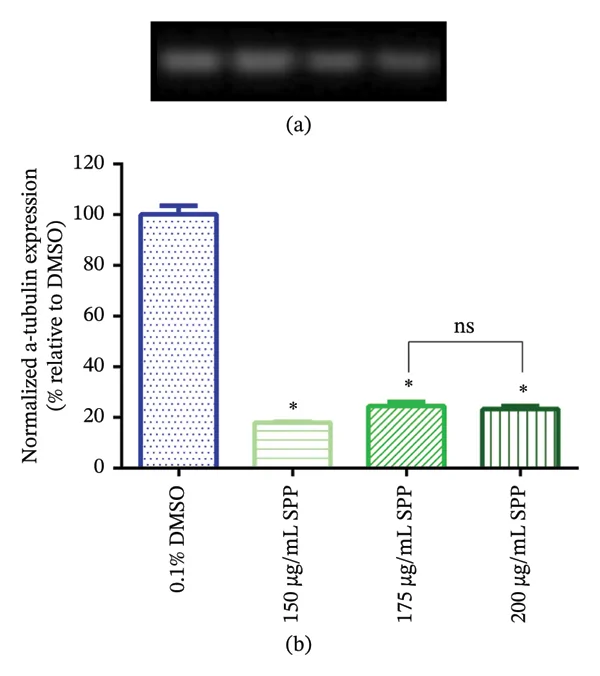
Soursop polyphenols (SPP) reduce *α*‐tubulin levels in *Giardia lamblia* trophozoites. (a) Representative western blot showing *α*‐tubulin expression in trophozoites after 48 h of exposure. Lane 1 corresponds to the DMSO‐treated control, while lanes 2, 3, and 4 correspond to treatments with 150, 175, and 200 μg/mL of SPP, respectively. (b) Densitometric quantification of *α*‐tubulin levels normalized to total protein per lane. Data are expressed as mean ± standard deviation of three independent experiments (*n* = 3). Statistical analysis was performed using one‐way ANOVA followed by Tukey’s post hoc test. ^∗^ *p* < 0.05 versus DMSO.

To determine whether treatment affected the parasite cytoskeleton, *α*‐tubulin expression and cellular distribution were evaluated in *Giardia* trophozoites. To assess changes in *α*‐tubulin expression induced by SPP, Western blot analysis was performed in *G. lamblia* trophozoites after 48 h of treatment. Protein expression was normalized to total protein loading, and densitometric analysis was expressed as a percentage relative to DMSO control, which was set as 100%. Under these conditions, *α*‐tubulin expression levels of 17.97%, 24.41%, and 23.24% were observed at SPP concentrations of 150, 175, and 200 μg/mL, respectively. Statistical analysis indicated that the values obtained at 175 and 200 μg/mL were not significantly different from each other (Figure 6).

**FIGURE 7:**
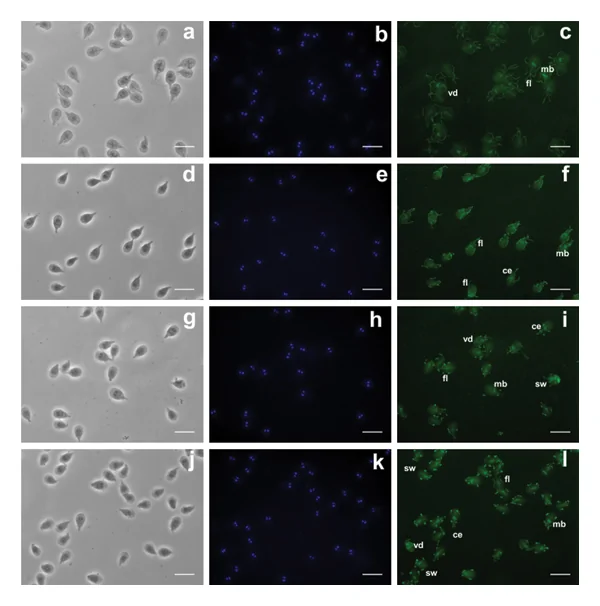
Soursop polyphenols (SPP) affect *α*‐tubulin distribution in *Giardia lamblia* trophozoites. Representative immunofluorescence micrographs show trophozoites exposed for 48 h to 0.1% DMSO (a–c) or SPP at 150 μg/mL (d–f), 175 μg/mL (g–i), and 200 μg/mL (j–l). *α*‐Tubulin is shown in green, and nuclei are stained with DAPI (blue). Cytoskeletal alterations include flagellar damage (fl), caudal elongation (ce), loss of the median body (mb), and ventral disk disorganization (vd). Swollen cells (sw) and punctate tubulin localization at flagellar tips were also observed. Scale bar = 20 μm.

To assess changes in *α*‐tubulin localization induced by SPP, *G. lamblia* trophozoites were analyzed by indirect immunofluorescence after 48 h of exposure. In control cells treated with 0.1% DMSO (Figure 7a–c), the characteristic pear shape and binucleate arrangement were preserved, with well‐defined tubulin labeling at the ventral disk (vd), median body (mb), and flagella (fl), consistent with typical cytoskeletal architecture.

In contrast, SPP‐treated cells exhibited marked alterations in both morphology and tubulin localization. At 150 μg/mL (Figure 7d–f), trophozoites showed elongation of the caudal region (ce), decreased fluorescence intensity in the median body and ventral disk, and frequent loss or shortening of flagella. Tubulin staining appeared fragmented or discontinuous in these structures. At higher concentrations (175 and 200 μg/mL; Figure 7g–l), more pronounced alterations were evident. Many cells appeared swollen (sw) or distorted, with diffuse or cytoplasmic tubulin staining. Notably, tubulin was often concentrated at the distal ends of residual flagella, forming punctate or dot‐like patterns in a concentration‐dependent manner. The ventral disk and median body were poorly defined or undetectable in many cells.

## 4. Discussion

The search for new antigiardial compounds remains an urgent priority due to the absence of a vaccine and the increasing occurrence of therapeutic failure. Plant‐derived extracts represent a promising alternative, with potential preventive, monotherapy, or adjuvant effects against *G. lamblia*, both in vitro and in vivo [5, 47–49]. Among the main advantages of these extracts is their potentially lower risk of inducing resistance, which is associated with the presence of multiple bioactive compounds with diverse mechanisms of action [50, 51].

Species of the *Annona* genus have a long history of use in traditional medicine for treating gastrointestinal ailments in Mexico. Previous studies by Calzada and collaborators demonstrated the antiprotozoal activity of *A. cherimola* and *A. muricata* against *G. lamblia* and *E. histolytica*. In particular, ethanol leaf extracts of *A. muricata* inhibited *G. lamblia* growth with an IC_50_ of 133.7 μg/mL (log_10_ IC_50_ ≈ 2.13), and nicotiflorin, kaempferol, and cis‐reticulatacin were identified among the most active constituents [16, 52]. Similar antiparasitic effects have been reported against *Cryptosporidium parvum*, where *A. muricata* leaf extract showed favorable interactions with parasite lactate dehydrogenase in silico and reduced parasite burden in infected mice. [53]. In agreement with these reports, the polyphenol‐enriched extract evaluated in the present study reduced trophozoite growth and cell adhesion and induced apoptosis‐like cell death, supporting the potential of *A. muricata* as a source of antigiardial compounds.

The biological activity observed in this study may be associated with the polyphenolic composition of the extract, which included (+)‐catechin and glucosides of apigenin, kaempferol, and cyanidin. Several of these compounds have previously demonstrated antiparasitic activity against kinetoplastid protozoa. In particular, kaempferol derivatives such as kaempferol 3,7‐O‐diglucoside and kaempferol 3‐O‐galactoside 7‐O‐rhamnoside exhibit activity against epimastigote, promastigote, amastigote, and trypomastigote forms of *Trypanosoma cruzi* and *Leishmania* species. Flavonoid compounds induce vacuolization, cellular shrinkage, and the appearance of electron‐dense inclusions, suggesting alterations in cellular metabolism and structural integrity [54, 55].

Among the potential molecular targets of flavonoids in protozoa, aldose reductase has recently attracted attention. Aldose reductase is a cytosolic enzyme involved in glucose metabolism, redox homeostasis, and osmoregulation, and it has also been implicated in the regulation of tubulin dynamics [29]. In mammalian systems and other protozoa, several flavonoids have been reported to modulate aldose reductase activity [30, 56–58]. Furthermore, a previous in silico study identified favorable interactions between kaempferol and *Giardia* aldose reductase, suggesting that this enzyme could represent a relevant pharmacological target in the parasite [32].

Consistent with these observations, all evaluated polyphenols were predicted to interact within the catalytic pocket of *Giardia aldose* reductase, overlapping the binding region of the reference inhibitor IDD‐594. Notably, the interactions involved residues Y40 and K71, which correspond to the conserved catalytic residues Y48 and K77 of human aldose reductase that participate in substrate reduction and cofactor stabilization [59, 60]. The docking results therefore support the possibility that the identified polyphenols may interact with aldose reductase and potentially interfere with its function.

Evidence from *Giardia* further supports a relationship between aldose reductase inhibition and structural alterations. Argüello‐García et al. reported that kaempferol‐induced inhibition of aldose reductase was associated with ultrastructural damage in *G. lamblia*, including multilamellar structures, enlarged perinuclear and periplasmic spaces, cytoplasmic alterations, elongated trophozoite forms, and electron‐dense granule deposition [32]. Although the specific morphological changes vary among protozoan species, flavonoids have consistently been associated with ultrastructural damage [54, 55]. The morphological abnormalities observed in the present study are compatible with previously described ultrastructural responses to metabolic and cytoskeletal disruption in protozoan parasites, including alterations in cell shape, flagellar organization, and intracellular architecture [61–63].

In addition to the metabolic effects associated with aldose reductase inhibition, several reports suggest that flavonoids can directly affect microtubule organization. Cyanidin‐3‐O‐glucoside disrupts microtubule‐associated processes in hepatocarcinoma cells through modulation of tubulin‐related proteins [64], whereas epigallocatechin‐3‐gallate interacts near the colchicine‐binding site of *α*‐tubulin and inhibits microtubule polymerization [65]. In *Giardia*, kaempferol exposure induces significant ultrastructural alterations, further supporting the ability of flavonoids to disrupt parasite cellular architecture [32].

A functional connection between aldose reductase activity and microtubule organization has also been reported. Aldose reductase can interact directly with tubulin, and alterations in glucose metabolism influence tubulin polymerization dynamics [66]. Moreover, oxidative stress in *Giardia* affects tubulin acetylation and polymerization, resulting in defects in the ventral disk and axonemal structures [36, 67]. Taken together, these observations suggest that metabolic disruption and cytoskeletal alterations may be mechanistically linked. The reduction in *α*‐tubulin expression and the changes in its intracellular distribution observed after SPP exposure are consistent with this hypothesis. Although the present study does not demonstrate a direct interaction between the polyphenols and tubulin, the combined evidence suggests that interference with aldose reductase activity may contribute to downstream alterations in microtubule organization. Additional biochemical studies will be required to determine whether tubulin is affected directly, indirectly, or through multiple convergent pathways.

Another factor that may contribute to the observed activity is the presence of multiple polyphenols acting simultaneously. Synergistic or additive interactions among plant‐derived phenolic compounds have been reported in numerous biological systems [68–71]. Similarly, catechin, apigenin, and kaempferol have demonstrated multitarget effects when combined with other phenolic compounds or antimicrobial agents [72–74]. Although these interactions were not directly evaluated in the present work, the antigiardial activity of the extract may result from the simultaneous modulation of multiple cellular targets.

Finally, the morphological and molecular alterations induced by SPP were accompanied by apoptosis‐like cell death. Similar effects have been reported in parasites treated with phenolic compounds [32, 41, 75–77]. Collectively, the available evidence suggests a multifactorial mechanism in which *A. muricata* polyphenols may affect both metabolic and cytoskeletal processes, ultimately compromising trophozoite growth, cell adhesion, and viability. However, the relationship between aldose reductase inhibition and cytoskeletal disruption remains hypothetical and is currently supported by docking predictions. Further studies involving enzymatic assays, target validation, and in vivo models are required to define the relative contribution of each mechanism and assess their therapeutic relevance.

## 5. Conclusions

Plant‐derived compounds represent a promising source of novel antigiardial agents. In this study, a polyphenol‐enriched fraction from *A. muricata* leaves was chemically characterized, revealing content of hydrolyzable polyphenols and flavonoids. HPLC/MS analysis identified five major polyphenolic compounds, including glycosylated derivatives of apigenin, kaempferol, and cyanidin, as well as (+)‐catechin.

Biological assays demonstrated that SPP impair *G. lamblia* growth and cell adherence, induced marked morphological alterations, affect *α*‐tubulin distribution, and promote apoptosis‐like cell death. In parallel, molecular docking analyses predicted favorable interactions between the identified polyphenols and *Giardia* aldose reductase, suggesting that this enzyme may represent a potential molecular target.

Taken together, the chemical, biological, and computational findings suggest that the antigiardial activity of *A. muricata* polyphenols may involve multiple cellular processes, including metabolic and cytoskeletal alterations. These findings expand the current knowledge of the pharmacological potential of *A. muricata* polyphenols and support the continued investigation of plant‐derived polyphenols and aldose reductase as promising subjects for future antigiardial studies.

## Funding

This work was partially supported by the Secretaría de Ciencia, Humanidades, Tecnología e Innovación (SECIHTI, Mexico) through the Sistema Nacional de Investigadoras e Investigadores (SNII). Lissethe Palomo‐Ligas is a member of the SNII (Researcher ID/CVU: 589916).

## Conflicts of Interest

The authors declare no conflicts of interest.

## Supporting Information

Additional supporting information can be found online in the Supporting Information section.

## Supporting information


**Supporting Information** Figure S1. Representative HPLC/MS chromatogram of the polyphenol‐enriched fraction obtained from *Annona muricata* (soursop) leaf ethanol extract. Five major peaks were detected and assigned as follows: (1) Cyanidin 3,5‐O‐diglucoside; (2) (+)‐catechin; (3) kaempferol 3,7‐O‐diglucoside; (4) apigenin 6,8‐di‐C‐glucoside; and (5) kaempferol 3‐O‐galactoside 7‐O‐rhamnoside. Figure S2. Validation of the docking protocol by re‐docking, in orange, IDD‐594 into the active site of the aldose reductase (PDB: 2R24), in blue, docking of IDD‐594 by Autodock 4.2.6.

## Data Availability

The data that support the findings of this study are available from the corresponding author upon reasonable request.

## References

[1] Bogitsh B. J. , Carter C. E. , and Oeltmann T. N. , Chapter 5-Visceral Protistans II: Flagellates, 2019, Academic Press.

[2] Adam R. D. , Giardia Duodenalis: Biology and Pathogenesis, Clinical Microbiology Reviews. (2021) 34, no. 4, 10.1128/CMR.00024-19. PMC8404698 34378955

[3] Golami S. , Rahimi-Esboei B. , Mousavi P. , Marhaba Z. , Youssefi M. R. , and Rahimi M. T. , Survey on Efficacy of Chloroformic Extract of Artemisia Annua Against Giardia Lamblia Trophozoite and Cyst in Vitro, Journal of Parasitic Diseases. (2016) 40, no. 1, 88–92, 10.1007/s12639-014-0453-3. 27065604 PMC4815849

[4] Fauziah N. , Aviani J. K. , Agrianfanny Y. N. , and Fatimah S. N. , Intestinal Parasitic Infection and Nutritional Status in Children Under Five Years Old: A Systematic Review, Tropical Medicine and Infectious Disease. (2022) 7, no. 11, 10.3390/tropicalmed7110371. PMC9697828 36422922

[5] Arguello-Garcia R. , Leitsch D. , Skinner-Adams T. , and Ortega-Pierres M. G. , Drug Resistance in Giardia: Mechanisms and Alternative Treatments for Giardiasis, Advances in Parasitology. (2020) 107, 201–282, 10.1016/bs.apar.2019.11.003. 32122530

[6] Lalle M. and Hanevik K. , Treatment-Refractory Giardiasis: Challenges and Solutions, Infection and Drug Resistance. (2018) 11, 1921–1933, 10.2147/IDR.S141468. 30498364 PMC6207226

[7] Ticona J. C. , Bilbao-Ramos P. , Amesty A. et al., Flavonoids From Piper Species as Promising Antiprotozoal Agents Against Giardia Intestinalis. Structure-Activity Relationship and Drug-Likeness Studies, Pharmaceuticals. (2022) 15, no. 11, 10.3390/ph15111386. PMC9695682 36355559

[8] Hulverson M. A. , Michaels S. A. , Lee J. W. et al., Identification of Fungus-Derived Natural Products as New Antigiardial Scaffolds, Microbiology Spectrum. (2023) 11, no. 3, 10.1128/spectrum.00647-23. PMC10269678 37039683

[9] Peckova R. , Dolezal K. , Sak B. et al., Effect of Selected Indonesian Plants on Giardia Intestinalis in an Experimental in Vitro Model, BMC Complementary Medicine and Therapies. (2025) 25, no. 1, 10.1186/s12906-025-04755-8. PMC11760690 39849480

[10] Quilez A. M. , Fernandez-Arche M. A. , Garcia-Gimenez M. D. , and De la Puerta R. , Potential Therapeutic Applications of the Genus Annona: Local and Traditional Uses and Pharmacology, Journal of Ethnopharmacology. (2018) 225, 244–270, 10.1016/j.jep.2018.06.014. 29933016

[11] Gutierrez-Pinzon Y. , Martinez-Preciado A. H. , Velazquez-Lopez J. M. et al., Antimicrobial Potential of Nanomaterials Synthesized With Extracts From Annona Plants: A Review, Antibiotics (Basel). (2025) 14, no. 8, 10.3390/antibiotics14080748. PMC12382639 40867943

[12] Mutakin M. , Fauziati R. , Fadhilah F. N. , Zuhrotun A. , Amalia R. , and Hadisaputri Y. E. , Pharmacological Activities of Soursop (Annona muricata Lin.), Molecules. (2022) 27, no. 4, 10.3390/molecules27041201. PMC8878098 35208993

[13] Mohd Abd Razak M. R. , Afzan A. , Ali R. et al., Effect of Selected Local Medicinal Plants on the Asexual Blood Stage of Chloroquine Resistant Plasmodium Falciparum, BMC Complementary and Alternative Medicine. (2014) 14, no. 1, 10.1186/1472-6882-14-492. PMC4300612 25510573

[14] Leesombun A. , Boonmasawai S. , and Nishikawa Y. , Ethanol Extracts From Thai Plants Have Anti-Plasmodium and Anti-Toxoplasma Activities in Vitro, Acta Parasitologica. (2019) 64, no. 2, 257–261, 10.2478/s11686-019-00036-w. 30820881

[15] Vila-Nova N. S. , de Morais S. M. , Falcao M. J. et al., Different Susceptibilities of Leishmania Spp. Promastigotes to the Annona muricata Acetogenins Annonacinone and Corossolone, and the Platymiscium floribundum Coumarin Scoparone, Experimental Parasitology. (2013) 133, no. 3, 334–338, 10.1016/j.exppara.2012.11.025. 23232251 PMC5426951

[16] Calzada F. , Correa-Basurto J. , Barbosa E. , Mendez-Luna D. , and Yepez-Mulia L. , Antiprotozoal Constituents From Annona Cherimola Miller, a Plant Used in Mexican Traditional Medicine for the Treatment of Diarrhea and Dysentery, Pharmacognosy Magazine. (2017) 13, no. 49, 148–152, 10.4103/0973-1296.197636. 28216899 PMC5307900

[17] Murrieta-Dionicio U. , Calzada F. , Barbosa E. et al., Antiprotozoal Activity Against Entamoeba Hystolitica and Giardia Lamblia of Cyclopeptides Isolated From Annona Diversifolia Saff, Molecules. (2024) 29, no. 23, 10.3390/molecules29235636. PMC11643917 39683795

[18] Hartati R. , Rompis F. M. , Pramastya H. , and Fidrianny I. , Optimization of Antioxidant Activity of Soursop (Annona muricata L.) Leaf Extract Using Response Surface Methodology, Biomed Reports. (2024) 21, no. 5, 10.3892/br.2024.1854. PMC11411398 39301565

[19] Chowdhury A. R. , Sharma S. , Mandal S. , Goswami A. , Mukhopadhyay S. , and Majumder H. K. , Luteolin, an Emerging Anti-Cancer Flavonoid, Poisons Eukaryotic DNA Topoisomerase I, Biochemical Journal. (2002) 366, no. Pt 2, 653–661, 10.1042/BJ20020098. 12027807 PMC1222798

[20] Martinez-Castillo M. , Pacheco-Yepez J. , Flores-Huerta N. et al., Flavonoids as a Natural Treatment Against Entamoeba Histolytica, Frontiers in Cellular and Infection Microbiology. (2018) 8, 10.3389/fcimb.2018.00209. PMC6024094 29988403

[21] da Silva E. R. , Brogi S. , Lucon-Junior J. F. , Campiani G. , Gemma S. , and Maquiaveli C. D. C. , Dietary Polyphenols Rutin, Taxifolin and Quercetin Related Compounds Target Leishmania Amazonensis Arginase, Food & Function. (2019) 10, no. 6, 3172–3180, 10.1039/c9fo00265k. 31134235

[22] Carter N. S. , Stamper B. D. , Elbarbry F. et al., Natural Products That Target the Arginase in Leishmania Parasites Hold Therapeutic Promise, Microorganisms. (2021) 9, no. 2, 10.3390/microorganisms9020267. PMC7911663 33525448

[23] Terra L. L. , Campanati L. , and De Souza W. , Heterogeneity in the Sensitivity of Microtubules of Giardia Lamblia to the Herbicide Oryzalin, Parasitology Research. (2010) 107, no. 1, 47–54, 10.1007/s00436-010-1831-0. 20405146

[24] Sudhakar A. , Kamanna S. , Bojja M. , and Tatu U. , Proteomic Analysis of Giardia Lamblia and Trichomonas Vaginalis Flagella Reveal Unique Post-Translational Modifications in Tubulin That Provide Clues to Regulation of Their Motilities, Proteomics. (2021) 21, no. 20, 10.1002/pmic.202100004. 34558204

[25] Trejo-Soto P. J. , Aguayo-Ortiz R. , Yepez-Mulia L. , Hernandez-Campos A. , Medina-Franco J. L. , and Castillo R. , Insights into the Structure and Inhibition of Giardia intestinalis Arginine Deiminase: Homology Modeling, Docking, and Molecular Dynamics Studies, Journal of Biomolecular Structure and Dynamics. (2016) 34, no. 4, 732–748, 10.1080/07391102.2015.1051115. 26017138

[26] Ihara S. , Miyamoto Y. , Le C. H. Y. et al., Conserved Metabolic Enzymes as Vaccine Antigens for Giardiasis, PLoS Neglected Tropical Diseases. (2022) 16, no. 4, 10.1371/journal.pntd.0010323. PMC9037923 35468132

[27] Morales-Luna L. , Vazquez-Bautista M. , Martinez-Rosas V. et al., Fused Enzyme Glucose-6-Phosphate Dehydrogenase::6-Phosphogluconolactonase (G6pd::6pgl) as a Potential Drug Target in Giardia Lamblia, Trichomonas Vaginalis, and Plasmodium falciparum, Microorganisms. (2024) 12, no. 1, 10.3390/microorganisms12010112. PMC10819308 38257939

[28] Klotz C. , Leisering R. , Hagen K. D. et al., Arginine Metabolism Has a Pivotal Function for the Encystation of *Giardia duodenalis* , bioRxiv. (2025) 10.1101/2025.02.20.639220. PMC12810918 41505477

[29] Ferrell M. , Abendroth J. , Zhang Y. et al., Structure of Aldose Reductase From Giardia Lamblia, Acta Crystallographica Section F: Structural Biology and Crystallization Communications. (2011) 67, no. Pt 9, 1113–1117, 10.1107/S1744309111030879. 21904059 PMC3169411

[30] Jung H. A. , Moon H. E. , Oh S. H. , Kim B. W. , Sohn H. S. , and Choi J. S. , Kinetics and Molecular Docking Studies of Kaempferol and Its Prenylated Derivatives as Aldose Reductase Inhibitors, Chemico-Biological Interactions. (2012) 197, no. 2-3, 110–118, 10.1016/j.cbi.2012.04.004. 22543015

[31] Choung W. J. , Hwang S. H. , Ko D. S. et al., Enzymatic Synthesis of a Novel Kaempferol-3-O-Beta-D-Glucopyranosyl-(1⟶4)-O-Alpha-D-Glucopyranoside Using Cyclodextrin Glucanotransferase and Its Inhibitory Effects on Aldose Reductase, Inflammation, and Oxidative Stress, Journal of Agricultural and Food Chemistry. (2017) 65, no. 13, 2760–2767, 10.1021/acs.jafc.7b00501. 28300406

[32] Arguello-Garcia R. , Calzada F. , Garcia-Hernandez N. , Chavez-Munguia B. , and Velazquez-Dominguez J. A. , Ultrastructural and Proapoptotic-Like Effects of Kaempferol in Giardia duodenalis Trophozoites and Bioinformatics Prediction of Its Potential Protein Target, Memorias do Instituto Oswaldo Cruz. (2020) 115, 10.1590/0074-02760200127. PMC7577037 33111756

[33] Rivelli J. F. , Amaiden M. R. , Monesterolo N. E. et al., High Glucose Levels Induce Inhibition of Na,K-Atpase via Stimulation of Aldose Reductase, Formation of Microtubules and Formation of an Acetylated Tubulin/Na,K-Atpase Complex, International Journal of Biochemistry & Cell Biology. (2012) 44, no. 8, 1203–1213, 10.1016/j.biocel.2012.04.011. 22565168

[34] Santander V. S. , Campetelli A. N. , Monesterolo N. E. et al., Tubulin-Na^+^, K^+^-Atpase Interaction: Involvement in Enzymatic Regulation and Cellular Function, Journal of Cellular Physiology. (2019) 234, no. 6, 7752–7763, 10.1002/jcp.27610. 30378111

[35] Valdez-Guerrero D. , Sandra G.-E. , Jesus M. et al., Isolation of Polyphenols From Soursop (Annona muricata L.) Leaves Using Green Chemistry Techniques and Their Anticancer Effect, Brazilian Archives of Biology and Technology. (2021) 64, 10.1590/1678-4324-2021200163.

[36] Palomo-Ligas L. , Estrada-Camacho J. , Garza-Ontiveros M. et al., Polyphenolic Extract From Punica granatum Peel Causes Cytoskeleton-Related Damage on Giardia Lamblia Trophozoites in Vitro, PeerJ. (2022) 10, 10.7717/peerj.13350. PMC9055998 35502204

[37] Garza-Ontiveros M. , Vargas-Villanueva J. R. , Gutiérrez-Gutiérrez F. et al., In Silico and in Vitro Antigiardiasic Potential of Grape Pomace Polyphenols Extracted by Hybrid Microwave-Ultrasound Methodology, Revista Brasileira de Farmacognosia. (2024) 34, no. 2, 313–327, 10.1007/s43450-023-00486-4.

[38] Aguirre-Garcia Y. L. , Castillo-Manzanares A. , Palomo-Ligas L. et al., Toxicity Evaluation of a Polyphenolic Extract From Flourensia cernua Dc Through Artemia Lethality Assay, Hemolytic Activity, and Acute Oral Test, Journal of Toxicology. (2024) 2024, no. 1, 10.1155/2024/2970470. PMC11329308 39157775

[39] Ascacio-Valdes J. A. , Aguilera-Carbo A. F. , Buenrostro J. J. , Prado-Barragan A. , Rodriguez-Herrera R. , and Aguilar C. N. , The Complete Biodegradation Pathway of Ellagitannins by Aspergillus Niger in Solid-State Fermentation, Journal of Basic Microbiology. (2016) 56, no. 4, 329–336, 10.1002/jobm.201500557. 26915983

[40] Keister D. B. , Axenic Culture of Giardia Lamblia in Tyi-S-33 Medium Supplemented With Bile, Transactions of the Royal Society of Tropical Medicine and Hygiene. (1983) 77, no. 4, 487–488, 10.1016/0035-9203(83)90120-7. 6636276

[41] Vargas-Villanueva J. R. , Gutierrez-Gutierrez F. , Garza-Ontiveros M. et al., Tubulin as a Potential Molecular Target for Resveratrol in Giardia Lamblia Trophozoites, in Vitro and in Silico Approaches, Acta Tropica. (2023) 248, 10.1016/j.actatropica.2023.107026. 37722447

[42] Hanwell M. D. , Curtis D. E. , Lonie D. C. , Vandermeersch T. , Zurek E. , and Hutchison G. R. , Avogadro: An Advanced Semantic Chemical Editor, Visualization, and Analysis Platform, Journal of Cheminformatics. (2012) 4, no. 1, 10.1186/1758-2946-4-17. PMC3542060 22889332

[43] Gasteiger J. and Marsili M. , A New Model for Calculating Atomic Charges in Molecules, Tetrahedron Letters. (1978) 19, no. 34, 3181–3184, 10.1016/S0040-4039(01)94977-9.

[44] Morris G. M. , Huey R. , Lindstrom W. et al., Autodock4 and Autodocktools4: Automated Docking With Selective Receptor Flexibility, Journal of Computational Chemistry. (2009) 30, no. 16, 2785–2791, 10.1002/jcc.21256. 19399780 PMC2760638

[45] Gutierrez-Gutierrez F. , Palomo-Ligas L. , Hernandez-Hernandez J. M. et al., Curcumin Alters the Cytoskeleton and Microtubule Organization on Trophozoites of Giardia Lamblia, Acta Tropica. (2017) 172, 113–121, 10.1016/j.actatropica.2017.04.027. 28465123

[46] Westerberg L. J. S. , Dedic B. , Naslund E. , Thorell A. , and Spalding K. L. , Superior Normalization Using Total Protein for Western Blot Analysis of Human Adipocytes, PLoS One. (2025) 20, no. 7, 10.1371/journal.pone.0328136. PMC12282925 40694567

[47] Abd-Elhamid T. H. , Abdel-Rahman I. A. M. , Mahmoud A. R. et al., A Complementary Herbal Product for Controlling Giardiasis, Antibiotics (Basel). (2021) 10, no. 5, 10.3390/antibiotics10050477. PMC8143091 33919165

[48] de Almeida C. R. , Bezagio R. C. , Colli C. M. , Romera L. I. L. , Ferrari A. , and Gomes M. L. , Elimination of Giardia duodenalis Biv in Vivo Using Natural Extracts in Microbiome and Dietary Supplements, Parasitology International. (2022) 86, 10.1016/j.parint.2021.102484. 34688884

[49] Alanazi A. D. , Combination Therapy of Green Synthesized Copper Nanoparticle and Metronidazole Showed Promising Efficacy Against Giardia Lamblia Infection, Acta Tropica. (2025) 272, 10.1016/j.actatropica.2025.107914. 41248727

[50] Alnomasy S. , Al-Awsi G. R. L. , Raziani Y. et al., Systematic Review on Medicinal Plants Used for the Treatment of Giardia Infection, Saudi Journal of Biological Sciences. (2021) 28, no. 9, 5391–5402, 10.1016/j.sjbs.2021.05.069. 34466120 PMC8381067

[51] Ozel Y. , Cavus I. , Unlu G. , Unlu M. , and Ozbilgin A. , Investigation of the Antitrichomonal Activity of Cinnamaldehyde, Carvacrol and Thymol and Synergy With Metronidazole, Turkiye Parazitoloji Dergisi. (2024) 48, no. 2, 72–76, 10.4274/tpd.galenos.2024.91855. 38958374

[52] Calzada F. , Merlin-Lucas V. I. , Valdes M. et al., Secondary Metabolites and Biological Properties of Annona muricata, Revista Brasileira de Farmacognosia. (2020) 30, no. 2, 305–311, 10.1007/s43450-020-00012-w.

[53] El-Wakil E. S. , Abdelmaksoud H. F. , Wakid M. H. et al., Annona muricata Leaf as an Anti-Cryptosporidial Agent: An in Silico Molecular Docking Analysis and in Vivo Studies, Pharmaceuticals. (2023) 16, no. 6, 10.3390/ph16060878. PMC10303196 37375825

[54] Marin C. , Ramirez-Macias I. , Lopez-Cespedes A. et al., In Vitro and in Vivo Trypanocidal Activity of Flavonoids From Delphinium Staphisagria Against Chagas Disease, Journal of Natural Products. (2011) 74, no. 4, 744–750, 10.1021/np1008043. 21466157

[55] Ramirez-Macias I. , Marin C. , Diaz J. G. , Rosales M. J. , Gutierrez-Sanchez R. , and Sanchez-Moreno M. , Leishmanicidal Activity of Nine Novel Flavonoids From Delphinium Staphisagria, The Scientific World Journal. (2012) 2012, 10.1100/2012/203646. PMC3366211 22666092

[56] Balestri F. , Poli G. , Pineschi C. et al., Aldose Reductase Differential Inhibitors in Green Tea, Biomolecules. (2020) 10, no. 7, 10.3390/biom10071003. PMC7407822 32640594

[57] Kannayiram K. , Umapathy V. R. , Chamundeswari Y. et al., Molecular Docking Analysis of Flavonoids With Aldose Reductase, Bioinformation. (2022) 18, no. 3, 80–83, 10.6026/97320630018180. 36518142 PMC9722430

[58] Yasir M. , Park J. , Han E. T. , Han J. H. , Park W. S. , and Chun W. , Investigating the Inhibitory Potential of Flavonoids Against Aldose Reductase: Insights From Molecular Docking, Dynamics Simulations, and Gmx_Mmpbsa Analysis, Current Issues in Molecular Biology. (2024) 46, no. 10, 11503–11518, 10.3390/cimb46100683. 39451563 PMC11506312

[59] Tarle I. , Borhani D. W. , Wilson D. K. , Quiocho F. A. , and Petrash J. M. , Probing the Active Site of Human Aldose Reductase. Site-Directed Mutagenesis of Asp-43, Tyr-48, Lys-77, and His-110, Journal of Biological Chemistry. (1993) 268, no. 34, 25687–25693, 10.1016/S0021-9258(19)74444-5. 8245005

[60] Walker E. H. , French C. E. , Rathbone D. A. , and Bruce N. C. , Mechanistic Studies of Morphine Dehydrogenase and Stabilization Against Covalent Inactivation, Biochemical Journal. (2000) 345, no. Pt 3, 687–692, 10.1042/bj3450687. 10642529 PMC1220805

[61] Rathore S. , Datta G. , Kaur I. , Malhotra P. , and Mohmmed A. , Disruption of Cellular Homeostasis Induces Organelle Stress and Triggers Apoptosis Like Cell-Death Pathways in Malaria Parasite, Cell Death and Disease. (2015) 6, no. 7, 10.1038/cddis.2015.142. PMC4650714 26136076

[62] Benchimol M. , Gadelha A. P. , and de Souza W. , Ultrastructural Alterations of the Human Pathogen Giardia Intestinalis After Drug Treatment, Pathogens. (2023) 12, no. 6, 10.3390/pathogens12060810. PMC10302959 37375500

[63] Morales-Luna L. , Hernandez-Ochoa B. , Gonzalez-Valdez A. et al., Nitazoxanide Analogs: Synthesis, in Vitro Giardicidal Activity, and Effects on Giardia Lamblia Metabolic Gene Expression, International Journal of Molecular Sciences. (2025) 26, no. 10, 10.3390/ijms26104504. PMC12111156 40429649

[64] Matboli M. , Hasanin A. H. , Hussein R. et al., Cyanidin 3-Glucoside Modulated Cell Cycle Progression in Liver Precancerous Lesion, in Vivo Study, World Journal of Gastroenterology. (2021) 27, no. 14, 1435–1450, 10.3748/wjg.v27.i14.1435. 33911466 PMC8047539

[65] Chakrabarty S. , Ganguli A. , Das A. , Nag D. , and Chakrabarti G. , Epigallocatechin-3-Gallate Shows Anti-Proliferative Activity in Hela Cells Targeting Tubulin-Microtubule Equilibrium, Chemico-Biological Interactions. (2015) 242, 380–389, 10.1016/j.cbi.2015.11.004. 26554336

[66] Rivelli J. F. , Ochoa A. L. , Santander V. S. , Nigra A. , Previtali G. , and Casale C. H. , Regulation of Aldose Reductase Activity by Tubulin and Phenolic Acid Derivates, Archives of Biochemistry and Biophysics. (2018) 654, 19–26, 10.1016/j.abb.2018.07.009. 30009780

[67] Patolsky R. G. , Laiolo J. , Diaz-Perez L. et al., Analysis of the Role of Acetylation in Giardia Lamblia and the Giardicidal Potential of Garcinol, Frontiers in Microbiology. (2024) 15, 10.3389/fmicb.2024.1513053. PMC11738946 39831116

[68] Pedrielli P. and Skibsted L. H. , Antioxidant Synergy and Regeneration Effect of Quercetin, (-)-Epicatechin, and (+)-Catechin on Alpha-Tocopherol in Homogeneous Solutions of Peroxidating Methyl Linoleate, Journal of Agricultural and Food Chemistry. (2002) 50, no. 24, 7138–7144, 10.1021/jf020437l. 12428973

[69] Szwajgier D. , Anticholinesterase Activity of Selected Phenolic Acids and Flavonoids-Interaction Testing in Model Solutions, Annals of Agricultural and Environmental Medicine. (2015) 22, no. 4, 690–694, 10.5604/12321966.1185777. 26706979

[70] Joshi T. , Deepa P. R. , and Sharma P. K. , Effect of Different Proportions of Phenolics on Antioxidant Potential: Pointers for Bioactive Synergy/Antagonism in Foods and Nutraceuticals, Proceedings of the National Academy of Sciences, India-Section B: Biological Sciences. (2022) 92, no. 4, 939–946, 10.1007/s40011-022-01396-6. 35935740 PMC9340677

[71] Liu H. , Xiang T. , Zang Q. et al., Synergy Analysis of Cyanidin-3-O-Glucoside and Catechin: Absorption, Transport and Lipid Metabolism Effects, Frontiers in Nutrition. (2025) 12, 10.3389/fnut.2025.1637637. PMC12402003 40904782

[72] Alvarez M. A. , Debattista N. B. , and Pappano N. B. , Antimicrobial Activity and Synergism of Some Substituted Flavonoids, Folia Microbiologica. (2008) 53, no. 1, 23–28, 10.1007/s12223-008-0003-4. 18481214

[73] Lehane A. M. and Saliba K. J. , Common Dietary Flavonoids Inhibit the Growth of the Intraerythrocytic Malaria Parasite, BMC Research Notes. (2008) 1, 10.1186/1756-0500-1-26. PMC2518919 18710482

[74] Sanhueza L. , Melo R. , Montero R. , Maisey K. , Mendoza L. , and Wilkens M. , Synergistic Interactions Between Phenolic Compounds Identified in Grape Pomace Extract With Antibiotics of Different Classes Against Staphylococcus aureus and Escherichia coli, PLoS One. (2017) 12, no. 2, 10.1371/journal.pone.0172273. PMC5325233 28235054

[75] Pais-Morales J. , Betanzos A. , Garcia-Rivera G. , Chavez-Munguia B. , Shibayama M. , and Orozco E. , Resveratrol Induces Apoptosis-Like Death and Prevents in Vitro and in Vivo Virulence of Entamoeba histolytica, PLoS One. (2016) 11, no. 1, 10.1371/journal.pone.0146287. PMC4701480 26731663

[76] Ali R. , Tabrez S. , Akand S. K. et al., Sesamol Induces Apoptosis-Like Cell Death in Leishmania donovani, Frontiers in Cellular and Infection Microbiology. (2021) 11, 10.3389/fcimb.2021.749420. PMC8581470 34778106

[77] Le H. G. , Kang J. M. , Vo T. C. , and Na B. K. , Kaempferol Induces Programmed Cell Death in Naegleria Fowleri, Phytomedicine. (2023) 119, 10.1016/j.phymed.2023.154994. 37597363

