# Supplementary material for: Annona muricata Polyphenols Induce Cytoskeletal Alterations and Predicted Aldose Reductase Binding in Giardia lamblia
Source: Adv Pharmacol Pharm Sci. 2026 Aug 25;2026:4133207. doi: 10.1155/adpp/4133207 (PMC13507698; doi:10.1155/adpp/4133207)
Supplement: Supplementary file 1 [file ADPP-2026-4133207-s001.docx]

**SUPPLEMENTARY MATERIALS**

***Annona muricata Polyphenols Induce Cytoskeletal Alterations and Predicted Aldose Reductase Binding in Giardia lamblia***

Govea-Flores Diana Valeria^1^ (ORCID 0009-0002-6399-5856), d.govea@uadec.edu.mx

Garza-Ontiveros Mariana^1^ (ORCID 0000-0002-6932-7090), garzamariana@uadec.edu.mx

Gutiérrez-Gutiérrez Filiberto^2^ (ORCID 0000-0001-9243-1390), filiberto.gutierrez@academicos.udg.mx

Gómez-Ibarra Ana Gabriela^1^ (ORCID 0009-0002-1924-4729), gabrielagomez@uadec.edu.mx

Nery-Flores Sendar Daniel^1^ (ORCID 0000-0003-0739-2230), sendar.nery@uadec.edu.mx

Ascacio-Valdés Juan Alberto^1^ (ORCID 0000-0001-6595-863X), alberto_ascaciovaldes@uadec.edu.mx

Campos-Múzquiz Lizeth Guadalupe^1^ (ORCID 0000-0002-7507-0885), l_campos@uadec.edu.mx

Rodríguez-Herrera Raúl^1^ (ORCID 0000-0002-6428-4925), raul.rodriguez@uadec.edu.mx

Palomo-Ligas Lissethe^1^* (ORCID 0000-0002-6399-5856), lissethe_palomo@uadec.edu.mx

*^1^Faculty of Chemical Sciences, Autonomous University of Coahuila, Saltillo Campus, Saltillo, Coahuila 25280, Mexico.*

*^2^Department of Pharmacobiology, University Center for Exact Sciences and Engineering, University of Guadalajara, Guadalajara, Jalisco 44430, Mexico*

***Supplementary Figure 1.*** *Representative HPLC/MS chromatogram of the polyphenol-enriched fraction obtained from Annona muricata (soursop) leaf ethanol extract. Five major peaks were detected and assigned as follows: (1) Cyanidin 3,5-O-diglucoside, (2) (+)-catechin, (3) kaempferol 3,7-O-diglucoside, (4) apigenin 6,8-di-C-glucoside, and (5) kaempferol 3-O-galactoside 7-O-rhamnoside.*

***Supplementary Figure 2:*** *Validation of docking protocol by re-docking, in orange, IDD-594 into the active site of the aldose reductase (PDB: 2R24), in blue, docking of IDD-594 by Autodock.4.2.6.*
